# Sirolimus treatment for intractable lymphatic anomalies: an open-label, single-arm, multicenter, prospective trial

**DOI:** 10.3389/fmed.2024.1335469

**Published:** 2024-02-08

**Authors:** Michio Ozeki, Saori Endo, Shiho Yasue, Akifumi Nozawa, Ryuta Asada, Akiko M. Saito, Hiroya Hashimoto, Takumi Fujimura, Yohei Yamada, Tatsuo Kuroda, Shigeru Ueno, Shoji Watanabe, Shunsuke Nosaka, Mikiko Miyasaka, Akihiro Umezawa, Kentaro Matsuoka, Takanobu Maekawa, Satoshi Hirakawa, Taizo Furukawa, Shigehisa Fumino, Tatsuro Tajiri, Junkichi Takemoto, Ryota Souzaki, Yoshiaki Kinoshita, Akihiro Fujino

**Affiliations:** ^1^Department of Pediatrics, Graduate School of Medicine, Gifu University, Gifu, Japan; ^2^Clinical Research Center, National Hospital Organization Nagoya Medical Center, Nagoya, Japan; ^3^Innovative and Clinical Research Promotion Center, Graduate School of Medicine, Gifu University, Gifu, Japan; ^4^Core Laboratory, Nagoya City University Graduate School of Medical Sciences, Nagoya, Japan; ^5^Department of Pediatric Surgery, Keio University School of Medicine, Tokyo, Japan; ^6^Department of Pediatric Surgery, Tokai University School of Medicine, Hiratsuka, Japan; ^7^Department of Plastic Surgery, Saitama Children’s Medical Center, Saitama, Japan; ^8^Department of Radiology, National Center for Child Health and Development, Tokyo, Japan; ^9^National Center for Child Health and Development, Research Institute, Tokyo, Japan; ^10^Department of Pathology, Tokyo Metropolitan Children’s Medical Center, Tokyo, Japan; ^11^Department of General Pediatrics and Interdisciplinary Medicine, National Center for Child Health and Development, Tokyo, Japan; ^12^Department of Dermatology, Hamamatsu University School of Medicine, Shizuoka, Japan; ^13^Department of Pediatric Surgery, Kyoto Prefectural University of Medicine, Kyoto, Japan; ^14^Department of Pediatric Surgery, Developmental Surgery and Intestinal Transplant Surgery, Kyushu University Hospital, Fukuoka, Japan; ^15^Department of Pediatric Surgery, Niigata University Medical and Dental Hospital, Niigata, Japan; ^16^Division of Surgery, Department of Surgical Subspecialties, National Center for Child Health and Development, Tokyo, Japan

**Keywords:** lymphatic anomalies, lymphatic malformation, generalized lymphatic anomaly, Gorham–stout disease, mammalian target of rapamycin

## Abstract

**Introduction:**

Intractable lymphatic anomalies (LAs) include cystic lymphatic malformation (LM; macrocystic, microcystic, or mixed), generalized lymphatic anomaly, and Gorham–Stout disease. LAs can present with severe symptoms and poor prognosis. Thus, prospective studies for treatments are warranted. We conducted a prospective clinical trial of sirolimus for intractable LAs.

**Methods:**

This was an open-label, single-arm, multicenter, prospective trial involving five institutions in Japan. All patients with LAs received oral sirolimus once daily, and the dose was adjusted to ensure that the trough concentration remained within 5–15 ng/mL. We prospectively assessed the drug response (response rate for radiological volumetric change in target lesion), performance state, change in respiratory function, visceral impairment (pleural effusion, ascites, bleeding, pain), laboratory examination data, quality of life (QOL), and safety at 12, 24, and 52 weeks of administration.

**Results:**

Eleven patients with LAs (9 generalized lymphatic anomaly, 1 cystic LM, 1 Gorham–Stout disease) were treated with sirolimus, of whom 6 (54.5%; 95% confidence interval: 23.4–83.3%) demonstrated a partial response on radiological examination at 52 weeks of administration. No patients achieved a complete response. At 12 and 24 weeks of administration, 8 patients (72.7%) already showed a partial response. However, patients with stable disease showed minor or no reduction after 12 weeks. Adverse events, such as stomatitis, acneiform dermatitis, diarrhea, and fever, were common with sirolimus. Sirolimus was safe and tolerable.

**Conclusion:**

Sirolimus can reduce the lymphatic tissue volume in LAs and may lead to improvements in clinical symptoms and QOL.

## 1 Introduction

There are various types of lymphatic anomalies (LAs) that fall under the category of complex LAs (1). These include common lymphatic malformation (LM), generalized lymphatic anomaly (GLA), kaposiform lymphangiomatosis, Gorham–Stout disease (GSD), central conducting lymphatic anomaly, and others (2). These anomalies typically manifest during birth or early childhood, and rarely occur in adulthood. The clinical symptoms of LAs are influenced by factors such as their anatomical region, size, and degree of invasiveness. LAs can lead to severe conditions such as dyspnea, respiratory disorders, coagulation disorders, and bleeding. Challenging LA cases can be complicated to manage because of the location and high risk of recurrence. Conventional treatment options include surgical resection, sclerotherapy, and drainage; however, these methods have potential complications. Therefore, it is crucial to explore innovative pharmacological treatment strategies for effective management of these anomalies.

Recent genetic studies have revealed that LAs are caused by somatic activating mutations in genes such as *PIK3CA*, *NRAS*, *CBL*, *KRAS*, and *ARAF* (3). These disease-causing genes and their associated proteins are involved in signaling pathways, including the PI3K-AKT (protein kinase B) and RAS-mitogen-activated protein kinase (MAPK) pathways that have roles in lymphatic vessel development and growth in both normal tissue and LAs (3). Another study showed that rapamycin (sirolimus), a mammalian target of rapamycin (mTOR) inhibitor, can inhibit the excessive growth of lymphatic vessels in mouse models of LM driven by mutations of the *PIK3CA* gene (4). In clinical settings, sirolimus has emerged as a promising treatment for LAs and other vascular anomalies (5). Retrospective cohort studies demonstrated that 50–80% of LA patients treated with sirolimus experienced improvements in their clinical symptoms, such as pain, bleeding, and functional deficits, and a reduction in their lesion volume. However, it is important to note that most of these studies were retrospective (6).

We conducted a multicenter prospective study to investigate the impacts of sirolimus for intractable LAs. In this study, we measured the volume of LM lesions objectively and quantitatively using magnetic resonance imaging (MRI). Additionally, based on the natural history data and effectiveness data of sirolimus from clinical trials that used the same MRI assessment as ours, we statistically determined the sample size and prospectively evaluated the radiological effect of sirolimus. Herein, we report the results of this trial for sirolimus treatment of LAs.

## 2 Methods

### 2.1 Study objectives, design, and enrollment

We performed a clinical trial, designated *Sirolimus for Intractable Lymphatic Anomalies* (SILA), to assess the efficacy and safety of sirolimus in LAs. It was an open-label, single-arm, multicenter, prospective trial conducted at five institutions in Japan (Gifu University Hospital, Kyoto Prefectural University of Medicine, Kyushu University Hospital, National Center for Child Health and Development, and Keio University School of Medicine).

Patients who met the eligibility criteria and provided informed consent and assent were enrolled. Before enrollment, all patients were reviewed by a multi-institutional, multidisciplinary evaluation committee, which included individuals from pediatric surgery, pediatrics, and radiology departments. Clinical information, clinical photographs, and radiological findings were assessed. Although there are no globally established diagnostic criteria for cystic LM, GLA, or GSD, we used the diagnostic criteria used domestically in Japan for each disease (7, 8). The inclusion criteria were: >0.6 m^2^ of body surface area (BSA) and judged by the investigator to be able to take tablets; definite diagnosis of cystic LM, GLA, or GSD, and exclusion of other lymphatic diseases; presence of at least one target lesion (e.g., cystic LM, lymphedema) measurable on MRI; and presence of intractable and severe disorders and symptoms caused by the target disease (bleeding, chronic pain, recurrent cellulitis [>3 episodes/year], ulceration, visceral and/or bone involvement, potential effects on organ function including the eye, airway, and ear). The exclusion criteria were: Karnofsky performance status (PS) score ≤ 30 (≥10 years of age) or Lansky play-PS ≤30 (<10 years of age); dysfunction of the liver, renal, or cardiac systems; receipt of molecularly targeted drugs associated with the mTOR pathway, immunosuppressant drugs, or drugs that inhibit/induce CYP3A4 enzyme activity; active infections requiring systemic treatment; uncontrolled diabetes, hypertension, hyperlipidemia, chronic liver, kidney disease, or immunodeficiency conditions; and carrier status for hepatitis B and/or hepatitis C virus. Patients with contraindications to sirolimus and experience of therapies affecting assessment of the target lesions, including history of allergic reaction to sirolimus, receipt of surgery (resection, sclerotherapy, or endovascular treatment), or administration of therapeutic drugs (propranolol, Kampo medicines, interferon, octreotide, bisphosphonate, or denosumab), chemotherapy agents, or radiation therapy for the target lesion, were also excluded.

### 2.2 Treatment and assessments

Initially, sirolimus (Rapalimus Tablet; 1 mg; Nobelpharma Co., Ltd., Tokyo, Japan) at 2 mg (BSA ≥1.0 m^2^) or 1 mg (BSA <1.0 m^2^) was administered orally once daily after meals or on an empty stomach. The dose was then adjusted based on the trough concentration of sirolimus measured during week 2 to reach a target concentration within 5 to 15 ng/mL (max dose: 4 mg). The trough concentrations were measured at 1 and 2 weeks, and every 4 weeks thereafter. Supportive therapies (antibiotics, blood transfusion, antipyretics, analgesics) and therapies that would not affect assessment of the LA lesions were allowed, while additional drugs that may have adverse effects in combination with sirolimus, drugs that inhibit/induce CYP3A4 enzyme activity, and therapies (surgery, sclerotherapy, and drugs) that may affect assessment of the target lesions were not allowed.

The primary outcome was the objective radiographic response rate to sirolimus treatment, defined as the population of patients who achieved a complete response (CR) or partial response (PR) based on the volume of the target lesion assessed on MRI at 52 weeks. For definitive evaluation, two central radiologists who were independent from the other investigators performed the assessment. On MRI with T2 fat-saturated sequence images of the lymphatic lesions or cysts, the area dimensions of the lesions were measured using the region of interest (ROI) tool in a Digital Imaging and Communications in Medicine (DICOM) viewer (OsiriX© v.9.0; Pixmeo, Bernex, Switzerland). Other pathologic lesions, such as inflammation, bleeding, and hematoma, were removed. The volume of the target lesion was calculated by multiplying the ROI areas by the slice width. The non-lymphatic tissues (bleeding, inflammation, and hematoma) were not examined. We classified the evaluation criteria as follows: CR, disappearance of all target lesions; PR, ≥20% decrease in volume of the target lesion; progressive disease (PD), ≥20% increase in volume of the target lesion; and stable disease (SD), insufficient shrinkage to qualify for PR and insufficient growth to qualify for PD.

The secondary outcomes were the radiological, clinical, and biological assessments of the efficacy of sirolimus as well as the safety. The radiological response rate (at 12 and 24 weeks), PS, change in respiratory function, visceral impairment (pleural effusion, ascites, bleeding, pain), laboratory examination data (complete blood count, liver, lipid profile, and blood coagulation parameters), and quality of life (QOL) were evaluated at 12, 24, and 52 weeks. The changes in vital signs and pharmacokinetic data every 4 weeks and the safety (adverse events) were examined during sirolimus treatment. PS, bleeding, pain, QOL, and adverse events were measured using the Karnofsky PS (≥10 years of age) or Lansky play-PS (<10 years of age), World Health Organization (WHO)-Bleeding Scale (9), visual analog scale for pain (10), PedsQL™ 4.0 Generic Core Scales (<25 years of age) (11) or Functional Assessment of Cancer Therapy-General (FACT-G) (≥25 years of age) (12), and the Common Terminology Criteria for Adverse Events V4.0 (13), respectively.

### 2.3 Sample size and data analysis

There is little available data on the natural history of intractable LAs. Therefore, we examined retrospective data from a Japanese national survey on intractable LAs (6). In 59 patients with intractable LAs (19 cystic LM, 18 GLA, and 22 GSD), only one patient (1.7%) showed radiological improvement for at least 1 year. Thus, the threshold response rate was 5%. The expected response rate was determined as 50% because data from past clinical trials showed radiological response rates of 52 and 50% (5, 14). Therefore, the sample size was calculated based on the probability that the lower limit of the exact 95% confidence interval (CI) for the primary outcome would be greater than the threshold of 5%. To maintain a statistical power of at least 90%, nine patients were needed. In consideration of drop-out, the target sample size was set at 10.

For the primary outcome, we used the full analysis set consisting of all enrolled patients and measured the response rate (proportion of patients evaluated as CR or PR) at 52 weeks and its exact 95% CI. If the lower limit of the 95% CI was >5%, efficacy of sirolimus would be concluded. For the secondary outcomes, the response rate and CI were calculated for pre-treatment versus 12, 24, and 52 weeks of treatment and compared by the Wilcoxon signed rank test if appropriate.

## 3 Results

**Figure 1:**
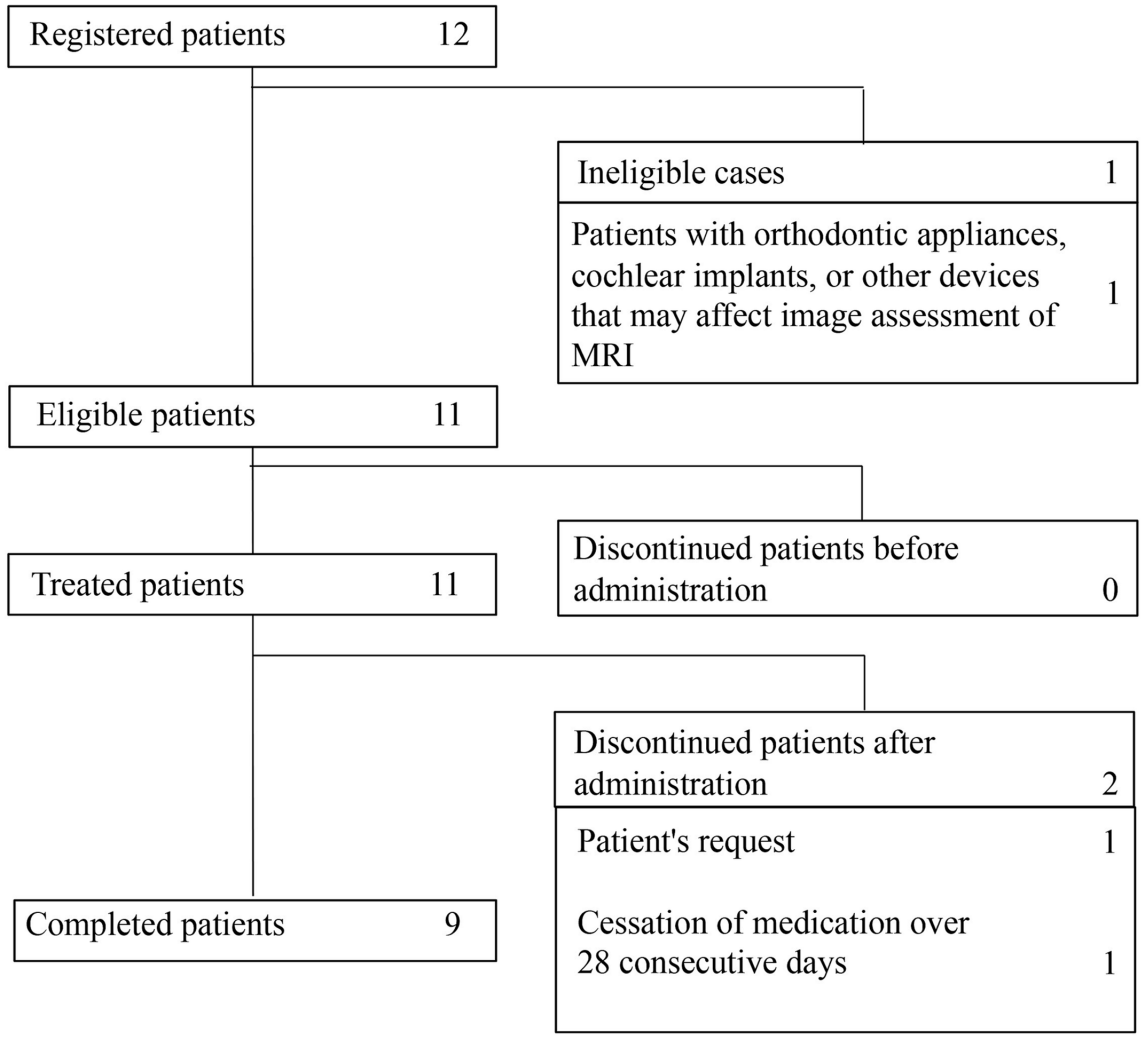
Patient distribution and inclusion in the analysis.

A total of 12 patients were enrolled in the study. Subsequently, 1 patient (case 9) was excluded because of an orthodontic appliance that may affect radiological assessment on MRI. Therefore, a total of 11 patients (9 GLA, 1 cystic LM, 1 GSD) were evaluated in the study. Of these, 2 patients did not complete the study because of discontinuation at the patient’s request and cessation of medication for >28 consecutive days during treatment, respectively (Figure 1).

The demographic characteristics of the patients treated with sirolimus (full analysis set) are shown in Table 1. All 11 patients had some complications (Table 1). The vital signs (blood pressure, heart rate, respiratory rate, body temperature, SpO_2_), electrocardiogram, and respiratory function tests of all patients before treatment were normal. Four patients (36.4%; cases 2, 3, 5, and 8) and 1 patient (9.1%; case 1) had pleural effusion and ascites. Cases 4 and 11 had lymphorrhea from the femoral skin. The target lesions for assessment comprised six subcutaneous lesions (cases 3, 6, 8, 10, 11, and 12), two retroperitoneal lesions (cases 1 and 4), two thoracic lesions (cases 2 and 5), and one cervical lesion (case 7). The mean drug-taking rate ± standard deviation was 97.3% ± 5.2%.

**Table 1:** Character of the patients treated with sirolimus (full analysis set).

Characteristics		Number (*n* = 11)
Sex (female/male)		7/4 (63.6%/36.4%)
Age at start of sirolimus		17.5 ± 9.6 (3–32)*
	<12	4 (36.4%)
	12–19	1 (9.1%)
	> = 20	6 (54.5%)
Height (cm)		143.1 ± 26.1 (98–176)*
Body weight (kg)		42.3 ± 22.36 (14.6–85.3)*
BMI (kg/m^2^)		19.19 ± 5.56 (14.9–33.3)*
BSA (m^2^)		1.275 ± 0.442 (0.63–1.95)*
Name of lymphatic anomaly	Cystic lymphatic malformation	1 (9.1%)
	Generalized lymphatic anomaly	9 (81.8%)
	Gorham-Stout disease	1 (9.1%)
Disease duration^#^ (year)		10.2 ± 7.6 (2–25)*
PS	Karnofsky PS score (≧10 years old) (*n* = 8)	86.3 ± 5.2 (80–90)*
	Lansky play-performance scale (<10 years old) (*n* = 3)	96.7 ± 5.8 (90–100)*
Sirolimus dose at 52 weeks or withdrawal	1 mg	1 (9.1%)
	2 mg	4 (36.4%)
	3 mg	3 (27.3%)
	4 mg	3 (27.3%)
Complications		
	Allergic rhinitis	4 (36.4%)
	Bleeding, food allergy, scoliosis, anemia and constipation	2 (18.2%)
	Others^+^	1 (9.1%)

*mean ± standard deviation (minimum-max). #duration from diagnosis to administration of sirolimus. +atopic dermatitis, Klippel-Trenaunay syndrome, gastroesophageal reflux disease, xeroderma, seasonal allergy, pleural effusion, coagulation factor deficiency, refractive amblyopia, conjunctivitis, thrombocytopenia, bone neoplasm, persistent left superior vena cava, ear neoplasm, heterotropia, congenital renal anomaly, ossification of the spinal ligaments, polycystic ovarian disease, cholecystolithiasis, albuminuria, hypofibrinogenemia, microsomia, pneumonorrhagia, rib fracture, urticaria, and decubitus ulcer. BMI, Body Mass Index; BSA, body surface area; PS, performance status.

### 3.1 Outcomes

**Figure 2:**
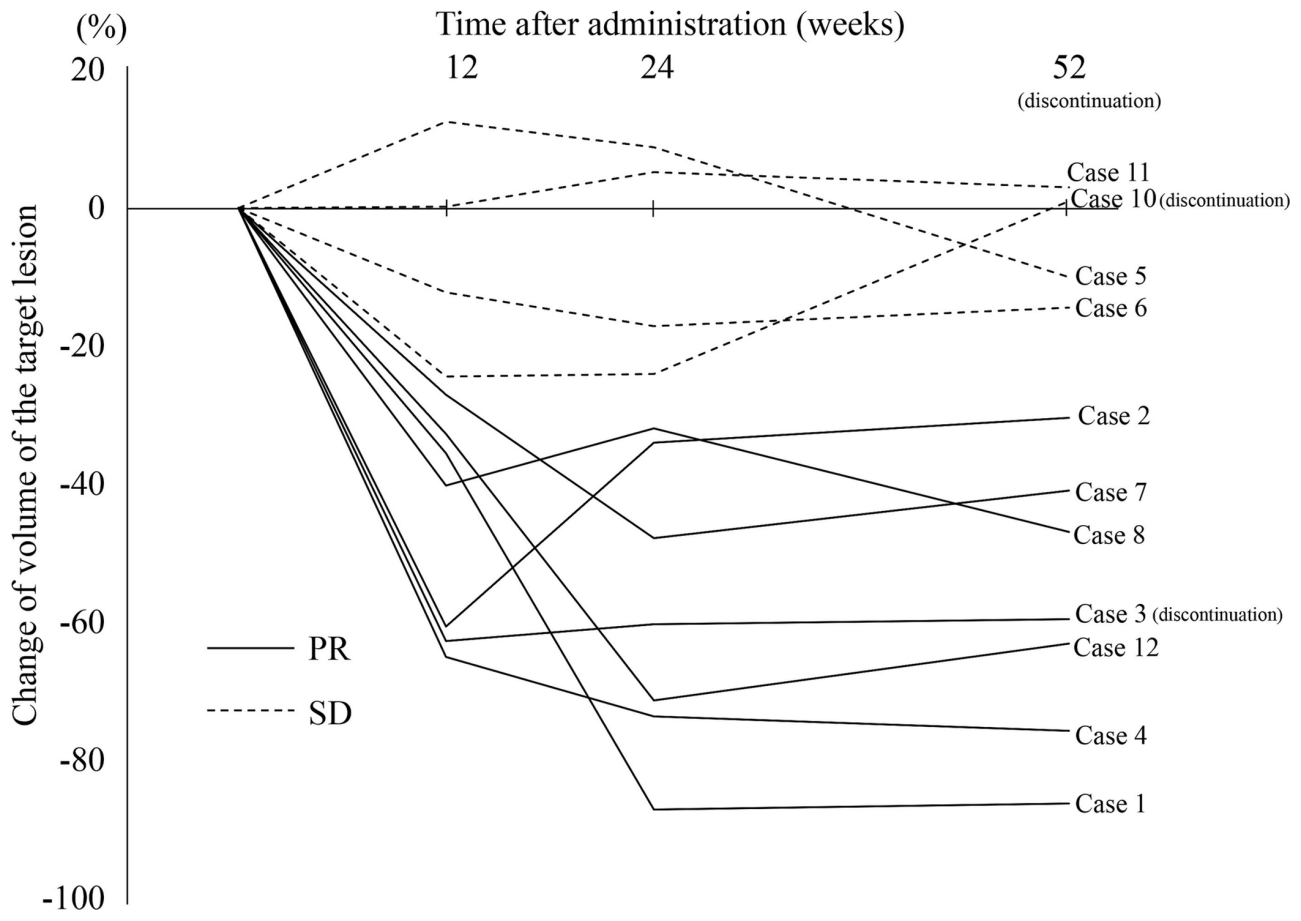
Changes in volume of the target lesions during sirolimus treatment. One patient (case 9) was excluded because of an orthodontic appliance that may affect radiological assessment on MRI. PR, partial response; SD, stable disease.

The detailed results for the individual cases are summarized in the Supplementary material. The radiological response rate at 52 weeks of administration was 54.5% (6/11; 95% CI: 23.4–83.3%; excluding case 3 who obtained PR but discontinued at day 287; Table 2). No patients achieved CR or PD. At 12 weeks of administration, 72.7% (8/11) already showed a PR and the volume of their lesions was further reduced at 24 weeks. However, in 7 PR patients (Figure 2, solid lines), the volume showed almost no change from 24 weeks to 52 weeks. In contrast, the SD patients (Figure 2, dotted lines) showed minor or no reduction after 12 weeks of treatment.

**Table 2:** Efficiency of sirolimus treatment after 12, 24 and 52 weeks or withdrawal.

	12 weeks		24 weeks		52 weeks or withdrawal	
	Number	(%)	Number	(%)	Number	(%)
	11		11		11	
CR	0	0.0	0	0.0	0	0.0
PR	8	72.7	8	72.7	7	63.6
SD	3	27.3	3	27.3	4	36.4
PD	0	0.0	0	0.0	0	0.0
Not evaluable	0	0.0	0	0.0	0	0.0
Efficiency (CR + PR)	8	72.7	8	72.7	6^†^	54.5
95% CI^‡^		[39.0, 94.0]		[39.0, 94.0]		[23.4, 83.3]
*p* value*						<0.001

CR, complete response; PR, partial response; SD, stable disease; PD, progressive disease; CI, confidential interval. ^†^Not included the withdrawal patient. ^‡^Calculated accurate confidence intervals based on binomial distribution. *One-sided test for the null hypothesis (response rate ≤ 5%).

Before the start of sirolimus treatment, pleural effusion was identified in 36.4% of patients (4/11). This proportion declined to 18.2% (2/11) at 52 weeks of treatment or discontinuation. Bleeding in the skin, soft tissues, muscles, and bones was observed in 4 of 11 patients before the treatment (Grade 1 in 2 cases and Grade 2 in 2 cases according to the WHO-Bleeding Scale). This number decreased to just one case (Grade 2) at 52 weeks of treatment or discontinuation. There were no instances of bleeding in the oral and nasal cavities, gastrointestinal tract, body cavities, central nervous system, points of surgical invasion, or circulatory system. Regarding the pain scales, no significant differences were noted before and after treatment. For the QOL scores, no significant changes were seen in the overall scores of the PedsQL (5 patients aged <25 years) or FACT-G (2 patients aged ≥26 years) between the pre-treatment period and 52 weeks of treatment. Likewise, there were no significant changes for improvement in activities of daily living (ADL) based on the pre-treatment and post-treatment Karnofsky PS scores (8 cases) and Lansky play-PS scores (3 cases).

Among the subjects with abnormal blood coagulation parameters at baseline, the rates of normalization at 52 weeks of treatment or discontinuation were as follows: 0.0% (0/3) for platelet count, 57.1% (4/7) for fibrinogen, and 20.0% (2/10) for D-dimer. While not all parameters became normalized, certain improvements were noted. The mean fibrinogen level increased after treatment, exceeding the baseline value at all observation points. Conversely, the mean D-dimer level decreased below the baseline value at all observation points after treatment commencement. No significant differences were observed before and after treatment (Table 3).

**Table 3:** Changes of clinical symptoms, blood coagulation test and QOL during sirolimus treatment.

	Pre-treatment	12 weeks	24 weeks	52 weeks	52 weeks or withdrawal
Pleural effusion	4/11 (36.4%), [10.9–69.2]^#^	4/11 (36.4%), [10.9–69.2]^#^	2/11 (18.2%), [2.3–51.8]^#^	1/9 (11.1%), [0.3–48.2]^#^	2/11 (18.2%), [2.3–51.8]^#^
Ascites	1/10 (10.0%), [0.3–44.5]^#^	0/9 (0%), [0–33.6]^#^	1/11 (9.1%), [0.2–41.3]^#^	2/9 (22.2%), [2.8–60.0]^#^	2/10 (20.0%), [2.5–55.6]^#^
QOL scores					
PedsQL (total scores)	93.5 (76–96)^+^ (*n* = 9)	86.9 (72–98)^+^ (*n* = 7)	84.8 (72–98)^+^ (*n* = 8)	93.5 (82–98)^+^ (*n* = 6)	92.4 (50–98) ^+^ (*n* = 8)
FACT-G (total scores)	71.5(68–75)^+^ (*n* = 2)	68.3 (68–69)^+^ (*n* = 2)	68.5 (65–72)^+^ (*n* = 2)	72.8 (72–74)^+^ (*n* = 2)	72.8 (72–74)^+^ (*n* = 2)
PS scores					
Karnofsky	80: 3/8 (37.5%) 90: 5/8 (62.5%) 100: 0/8 (0%)	80: 0/8 (0%) 90: 8/8 (100%) 100: 0/8 (0%)	80: 1/8 (12.5%) 90: 7/8 (87.5%) 100: 0/8 (0%)	80: 0/7 (0%) 90: 7/7 (100%) 100: 0/7 (0%)	80: 0/8 (0%) 90: 8/8 (100%) 100: 0/8 (0%)
Lansky	80: 0/3 (0%) 90: 1/3 (33.3%) 100: 2/3 (66.7%)	80: 0/3 (0%) 90: 2/3 (66.7%) 100: 1/3 (33.3%)	80: 0/3 (0%) 90: 2/3 (66.7%) 100: 1/3 (33.3%)	80: 0/2 (0%) 90: 0/2 (0%) 100: 2/2 (100%)	80: 0/3 (0%) 90: 1/3 (33.3%) 100: 2/3 (66.7%)
Blood coagulation test^*^					
Platelet (×10^4^/μL)	9.1 (6.5–49.4)^+^ (*n* = 3)	8.70 (5.5–32.3)^+^ (*n* = 3)	10.3 (6.3–10.6)^+^ (*n* = 3)	11.1 (6.5–15.6)^+^ (*n* = 2)	8.2 (6.5–15.6) ^+^ (*n* = 3)
Fibrinogen (mg/dL)	169.0 (74–416)^+^ (*n* = 7)	250.0 (111–465)^+^ (*n* = 7)	240.5 (201–419)^+^ (*n* = 6)	291.0 (137–350)^+^ (*n* = 5)	212.0 (137–350)^+^ (*n* = 7)
D-dimer (μg/mL)	14.2 (1.7–101.1)^+^ (*n* = 10)	8.9 (1.2–63.6)^+^ (*n* = 10)	3.6 (0.6–48.4)^+^ (*n* = 9)	5.4 (0.5–53.4)^+^ (*n* = 8)	6.95 (0.5–53.4)^+^ (*n* = 10)
Pain scale^*^					
	15.0 (0–76)^+^ (*n* = 10)	6.0 (0–69)^+^ (*n* = 10)	22.5 (0–64)^+^ (*n* = 10)	22.0 (0–55)^+^ (*n* = 8)	23.5 (0–55)^+^ (*n* = 10)

#[95% CI]. +median (minimum-max). *The data of patients whose levels of coagulation parameters and VAS before treatment were abnormal. CI, confidence interval; VAS, visual analog scale; QOL, quality of life; FACT-G, Functional Assessment of Cancer Therapy-General; PS, performance status.

We observed improvement of symptoms in three cases (cases 5, 6, and 11) that were assessed as SD (Supplementary material). In case 5, the pleural effusion observed before the start of treatment had disappeared by week 52 of treatment. The patient’s pre-treatment VAS score of 64 had improved to 23 at 52 weeks. In case 6, improvements were noted in the blood coagulation parameters: fibrinogen (74 mg/dL pre-treatment to 137 mg/dL at 52 weeks) and D-dimer (101.1 μg/mL pre-treatment to 53.4 μg/mL at 52 weeks). Although the volume change rate of the target lesion fell between −17.1 and − 12.2%, and the therapeutic effect did not reach PR, a discernible reduction was evident. In case 11, the pre-treatment VAS score of 76 improved to 24 at 52 weeks. Pre-treatment Grade 2 bleeding (involving the skin, soft tissue, muscle, bone) had resolved at 52 weeks. Before treatment, the patient had lymphatic leakage of 1–2 L/day, which progressively decreased up to 24 weeks until there was no leakage and the skin condition became softer.

### 3.2 Sirolimus treatment and drug concentrations

**Figure 3:**
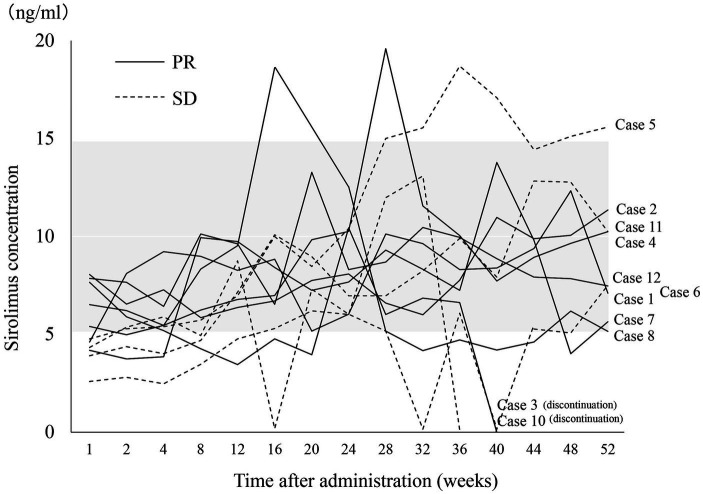
Changes in sirolimus concentrations during treatment. The gray band indicates the target trough sirolimus concentration range (5–15 ng/mL). One patient (case 9) was excluded because of an orthodontic appliance that may affect radiological assessment on MRI. PR, partial response; SD, stable disease.

The sirolimus trough levels were measured at 1 and 2 weeks, and then every 4 weeks thereafter. The target trough level was set between 5 to 15 ng/mL, and the dosage of sirolimus was adjusted accordingly. The sirolimus concentration at nearly all measurement points was within the target range (Figure 3). There were no notable differences in sirolimus concentrations between patients with PR and patients with SD. The sirolimus concentrations in all SD patients (cases 5, 6, 10, and 11) at 1 week and two SD patients (cases 6 and 11) at 2, 4, and 8 weeks were below the 5 ng/mL threshold. However, these low concentrations were not correlated with the effectiveness of sirolimus, and the concentrations in these patients varied throughout the rest of the treatment period.

### 3.3 Safety

Adverse events were observed in all 11 subjects, but none exceeded Grade 3 in severity. The Grade 3 adverse events were observed in 7 subjects (63.6%). There were no instances of fatal adverse events. Stomatitis was the most common adverse event, occurring in 9 patients (81.8%), followed by acneiform dermatitis in 8 (72.7%) and then diarrhea and fever in 6 subjects each (54.5%). Other observed events included upper respiratory infection in 4 patients (36.4%), and abdominal pain, pharyngitis, skin infection, and pain in 3 subjects each (27.3%). Eczema, bronchitis, pneumonia, influenza-like symptoms, dehydration, cough, arthropod bite, skin abrasion, muscle pain, headache, and heavy menstrual bleeding were observed in 2 subjects each (18.2%). All other adverse events were singular occurrences.

## 4 Discussion

Intractable LAs are extremely rare and have limited experience details and treatment options, making these diseases exceptionally difficult to manage. Historically, treatment strategies have primarily consisted of surgical intervention, sclerotherapy, and symptomatic care. However, there is an urgent need for novel treatments to address the significant challenge of managing these diseases. The present study is the first multicenter clinical trial in Japan designed to prospectively investigate the therapeutic efficacy and safety of oral sirolimus for intractable LAs. Sirolimus was administered for a 1-year period, with blood level monitoring after each administration to maintain a therapeutic concentration between 5 and 15 ng/mL. MRI was employed to assess the target lesions, with evaluations for reduction in lesion size, clinical symptoms, bleeding, blood coagulation test data, QOL, ADL, and any adverse events. The findings demonstrated that sirolimus can significantly reduce the lesion size and may lead to improvements in symptoms associated with LAs.

Sirolimus is postulated to exert its therapeutic effects through the inhibition of mTOR-related proteins, resulting in suppression of both angiogenesis and lymphangiogenesis (4). Recent advances in genetic analyses have uncovered somatic mosaic mutations of the *PIK3CA* gene within the tissues of LA lesion sites, casting a spotlight on the correlations between these mutations and the pathogenesis of LAs (2). Current understanding posits that mouse models with *PIK3CA* gene mutations within lymphatic endothelial cells are prone to the development of purulent lesions (3). It is further hypothesized that the presence of *PIK3CA* gene mutations in LAs could result in a reduction in lesion size, coupled with a potential decrease in lymph leakage from the lesions, after sirolimus treatment. Although the anatomical location of lymphatic malformations is important when making treatment choices, in this study we included cases that presented with severe symptoms, regardless of being in anatomically risky locations, because sirolimus has the potential these symptoms as well.

In the present study, we refrained from conducting genetic analyses on the lesion sites, and thus no cases were officially confirmed to have *PIK3CA* gene mutations. Nevertheless, the notable reductions in lesion size and the resulting clinical improvements observed across a wide range of cases suggest a strong correlation between LAs and *PIK3CA* gene mutations. Alternatively, even in the absence of *PIK3CA* gene mutations, the suppressive effects on angiogenesis and lymphangiogenesis could be key mechanisms driving the observed clinical improvements. Biopsy or removal of the lesion site carries risks of severe bleeding, infection, and postoperative lymph leakage. In clinical practice, it is not easy to perform genetic analysis before administering sirolimus treatment. Additionally, the detection sensitivity for genetic mutations in lesions is not currently high, and the process is time-consuming and costly. To date and at the time of the study’s planning, there is no established evidence linking genetic mutations to the effectiveness of sirolimus treatment. These insights challenge the conventional belief on the necessity to confirm the presence or absence of genetic mutations prior to commencement of treatment. Further research is needed to understand the relationship between the genetic background of the patient and the treatment efficacy of sirolimus.

We have conducted a prospective study that assessed not only the reduction rate of the target lesions, but also the clinical symptoms, pleural effusion, ascites, blood and coagulation test data, pain scales, QOL, ADL, and drug kinetics during treatment. These consecutive and comprehensive data were valuable, and we confirmed the efficacy of sirolimus in reducing the size of LA lesions. We analyzed the MRI images of the lesion sites in all cases using a volumetric approach. Through application of the image analysis software OsiriX, we precisely detected and measured high-signal lymphatic cysts and edema that were visible on fat-suppressed T2-weighted images. Because lymphatic disease lesions have complex shapes, their measurement presents considerable challenges. However, OsiriX allowed us to establish ROIs based on MRI signal intensity variations, thereby facilitating area measurement. This makes OsiriX a valuable tool for evaluation of lymphatic diseases. In addition, we evaluated a variety of parameters during treatment. A global project that involves numerous doctors and patients, known as Outcome Measures for Vascular Malformations (OVAMA) (15), also promotes and is developing the use of various parameters such as symptoms, QOL, and patient satisfaction for treatment evaluation. Our trial, which was initiated before the OVAMA project, implemented similar evaluations as part of a prospective study. Even in cases where the lesion size was not reduced, we often observed improvements in other clinical symptoms. Consequently, it is vital that relevant judgments are based on an all-encompassing view, employing a variety of evaluation criteria and not solely relying on MRI images.

The optimal concentration and duration of sirolimus for vascular anomalies remain unknown. In our study, we adjusted the dosage to yield a target trough concentration of 5–15 ng/mL. Throughout the treatment, the fluctuations of sirolimus concentrations were largely confined within our target trough concentration range. However, the concentration levels in all SD patients (cases 5, 6, 10, and 11) were < 5 ng/mL after 1 week, and for two of these patients (cases 6 and 11), the levels remained below 5 ng/mL at 2, 4, and 8 weeks and the drug dosage was increased. Harbers et al. (16) pointed out the effectiveness of low target sirolimus levels (4–10 ng/mL) in 79.1% of patients with vascular anomalies. Despite these findings, low concentrations are not universally optimal for all cases. Karastaneva et al. (17) reported that the most remarkable volume reductions were detected within the first 4–6 months of therapy. In all of our PR patients, the tumors had already reduced by >20% at 12 weeks, and a similar trend persisted at 24 and 52 weeks. It is important to mention that the link between treatment responsiveness and blood concentration remains elusive. Nevertheless, it is plausible that an initially low blood concentration may lead to subpar treatment outcomes. High blood levels of sirolimus (>10 ng/mL) can lead to severe adverse events (18, 19), suggesting that if effective outcomes can be achieved at lower concentrations, there is no justification for enforcing higher levels. In our view, if the efficacy is not immediately apparent after treatment initiation, it is advisable to adjust the dosage to reach the range of 5–15 ng/mL and persist with the treatment for at least 6 months.

The adverse events observed were considered to be of a similar type and frequency to those reported in previous studies (5, 14, 16, 17, 19). Regarding our female patients, we found that 3 of 7 (42.9%) experienced menstrual disorders (1 irregular menstruation, 2 hypermenorrhea). These patients maintained regular menstrual cycles before administration of sirolimus, and their symptoms proved to be mild and reversible. Similarly, Triana et al. (20) reported that 7 of 74 women (9.4%) experienced menstrual alterations attributed to sirolimus treatment. All of these patients also maintained regular menstrual cycles before the treatment. One patient experienced a 4-month amenorrhea period after treatment initiation, which later resolved spontaneously, and the other six patients encountered hypermenorrhea. While most patients experienced mild menstrual alterations that did not necessitate dose reduction or withdrawal, one patient had to discontinue sirolimus due to hypermenorrhea, metrorrhagia, and hematuria. Following sirolimus withdrawal, five patients saw their regular menstrual cycles return. Using a rat model, Braun et al. (21) investigated the underlying mechanisms for sirolimus-associated ovarian toxicity. They discovered that sirolimus amplified signaling in rat ovarian follicles via the pro-proliferative PI3K pathway. We should take care for these potential adverse events.

Our study had some limitations. First, the open-label, single-arm design may have introduced bias in some outcome measures. Ideally, a randomized, placebo-controlled, double-blind clinical trial design should be used. However, these diseases are extremely rare and heterogeneous, and thus the trials are challenging. A nationwide survey by Japanese rare disease researchers in the 2010s showed that intractable cases in Japan included about 600 with a cystic lymphatic malformation and 100 with GLA/GSD (7, 8). These numbers highlight the limited number of potential participants in Japan. Enrolling suitable cases within the trial period was challenging given few patients could reach the trial sites and meet the inclusion criteria. Therefore, conducting a high evidence level trial for such rare diseases is difficult. Furthermore, evaluation methods for vascular anomalies have not been established, and it is difficult to assess the efficacy of sirolimus treatment.

Second, the number of participants was small. We designed the sample size to allow assessment of the radiological response rate as the primary outcome. There has been no previous clinical trial of sirolimus with a sample size determined statistically. However, the sample size was not necessarily appropriate for the secondary outcomes. Despite improvements in clinical symptoms, it is possible that there were no significant changes in almost all secondary outcomes such as QOL. Furthermore, the age group was limited to those who could take sirolimus tablets orally, so infants were not included. Separate trials may be necessary for infants.

Third, severity and radiological evaluation are not always correlated. Therefore, the reduction of lesions observed in our clinical trial does not necessarily indicate an improvement in clinical symptoms. However, it is evident that sirolimus significantly reduced the lesions compared with their natural history. Previous studies reported that the reduction of LM was associated with improvements in clinical symptoms and QOL (14). We evaluated secondary outcomes, such as other symptoms and QOL measures. Unfortunately, the number of cases was too small for a conclusive assessment of each item. Regardless, increasing the number of cases would enhance the probability of the data; thus, further investigation of higher numbers of cases is necessary in the future.

In Japan, sirolimus has been designated as an orphan drug. The results of this study have led to its regulatory approval in Japan. We reported the outcomes of the clinical trial based on the maximum information available and the scientific perspective at the time of planning the study.

In conclusion, our results show that sirolimus appears to be effective for decreasing the lesion size in intractable LAs. Adverse events of sirolimus were common, but were mostly mild and tolerable if monitored carefully. Further clinical trials are warranted to investigate the optimal concentration and duration of sirolimus treatment.

## Data availability statement

The original contributions presented in the study are included in the article/Supplementary material, further inquiries can be directed to the corresponding author.

## Ethics statement

The studies involving humans were approved by Gifu University Institutional Review Board. The studies were conducted in accordance with the local legislation and institutional requirements. Written informed consent for participation in this study was provided by the participants’ legal guardians/next of kin.

## Author contributions

MO: Writing – original draft, Conceptualization, Funding acquisition, Investigation, Methodology, Project administration, Supervision. SE: Investigation, Methodology, Writing – review & editing. SY: Investigation, Methodology, Writing – review & editing. AN: Data curation, Investigation, Methodology, Writing – review & editing. RA: Conceptualization, Data curation, Funding acquisition, Methodology, Project administration, Writing – review & editing. AS: Data curation, Formal analysis, Methodology, Validation, Writing – review & editing. HH: Data curation, Formal analysis, Writing – review & editing. TkF: Investigation, Writing – review & editing. YY: Investigation, Writing – review & editing. TK: Investigation, Writing – review & editing. SU: Investigation, Methodology, Writing – review & editing. SW: Investigation, Methodology, Writing – review & editing. SN: Formal analysis, Investigation, Writing – review & editing. MM: Formal analysis, Investigation, Writing – review & editing. AU: Investigation, Methodology, Writing – review & editing. KM: Formal analysis, Investigation, Methodology, Writing – review & editing. TM: Conceptualization, Investigation, Methodology, Writing – review & editing. SH: Formal analysis, Investigation, Methodology, Writing – review & editing. TiF: Investigation, Methodology, Writing – review & editing. SF: Investigation, Writing – review & editing. TT: Investigation, Methodology, Writing – review & editing. JT: Investigation, Writing – review & editing. RS: Investigation, Writing – review & editing. YK: Investigation, Writing – review & editing. AF: Conceptualization, Investigation, Methodology, Writing – review & editing.
