# Supplementary material for: Sirolimus treatment for intractable lymphatic anomalies: an open-label, single-arm, multicenter, prospective trial
Source: Front Med (Lausanne). 2024 Feb 8;11:1335469. doi: 10.3389/fmed.2024.1335469 (PMC10881825; doi:10.3389/fmed.2024.1335469)
Supplement: Supplementary file 1 [file Data_Sheet_1.PDF]

Supplementary Material

Sirolimus treatment for intractable lymphatic anomalies (SILA):an open-label, single-arm, multicenter, prospective trial

Michio Ozeki*, Saori Endo, Shiho Yasue, Akifumi Nozawa, Ryuta Asada, Akiko M. Saito, Hiroya Hashimoto, Takumi Fujimura, Yohei Yamada, Tatsuo Kuroda, Shigeru Ueno, Shoji Watanabe, Shunsuke Nosaka, Mikiko Miyasaka, Akihiro Umezawa, Kentaro Matsuoka, Takanobu Maekawa, Satoshi Hirakawa, Taizo Furukawa, Shigehisa Fumino, Tatsuro Tajiri, Junkichi Takemoto, Ryota Souzaki, Yoshiaki Kinoshita, Akihiro Fujino

*** Correspondence:** Michio Ozeki: ozeki.michio.j5@f.gifu-u.ac.jp

1 Supplementary Data

1. Clinical data of each patient treated with sirolimus

a) case number, age (years old), sex, diagnosis, sites of lesion, height (cm), body weight (kg) and body surface area (m^2)

b) change of volume of the target lesion, evaluation of lesions and images of magnetic resonance imaging (MRI)

c) secondary assessments; pleural effusion, ascites, change of other clinical symptoms, blood coagulation test (platelet counts, fibrinogen and D-dimer), bleeding scales (the World Health Organization-Bleeding Scale), pain scales (visual analog scales), Functional Assessment of Cancer Therapy-General (FACT-G) (>25 years old)) and performance status (Karnofsky Performance Status (>10 years old), Lansky play-performance scale (for patients <10 years old), and Quality of Life (QOL) scores (PedsQL™ 4.0 Generic Core Scales (< 25 years old).

d) adverse events; events and grade (the Common Terminology Criteria for Adverse Events V4.0) divided into periods (after 0-12 weeks, 13-24 weeks, 25-52 weeks) (underline: causal relationship cannot be ruled out)

e) plasma sirolimus concentration (ng/ml) and drug dose (mg)

Case 1

a) 32 years old, female, GLA (pelvis and retroperitoneum), 156cm, 40.8kg, 1.33m^2

b)

	Pre-treatment	After 12 weeks	After 24 weeks	After 52 weeks
Change of volume of the target lesion		-35.5%	-87.1%	-86.2%
Evaluation of lesions		PR	PR	PR
MRI	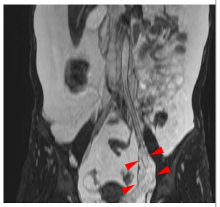	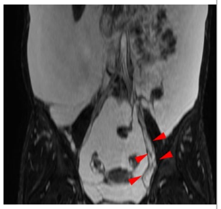	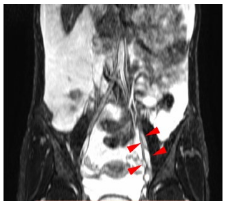	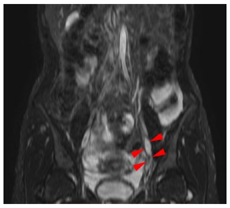
	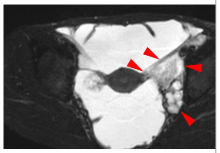	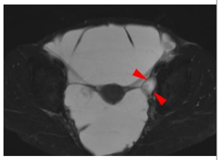	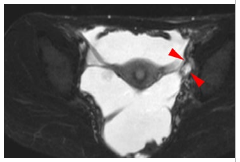	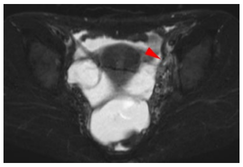

c)

	Pre-treatment	After 12 weeks	After 24 weeks	After 52 weeks
Pleural effusion	-	-	-	-
Ascites	+	N.A.	+ (decrease)	+ (decrease)
Change of other clinical symptoms		Improvement of abdominal distension		
Platelet counts (/ul)	24.6	23.7	21.5	21.9
Fibrinogen (mg/dl)	220	311	240	386
D-dimer (ug/ml)	6.8	1.2	0.6	<0.5
Bleeding scale	None	None	None	None

Pain scale	23	2	2	0
PS (Karnofsky)	80	90	90	90
QOL (FACT-G)	68.0	68.7	72.0	71.5

d)

	0-12 weeks	13-24 weeks	25-52 weeks
Events (CTCAE grade)	Irregular menstruation (2), headache (1), pharyngitis (2), stomatitis (2), constipation (1) and acne (1)	Disease resembling influenza (2)	Stomatitis (1), stomatitis (1), acne (1), stomatitis (1), acne (1) and upper respiratory infections (2)

e)

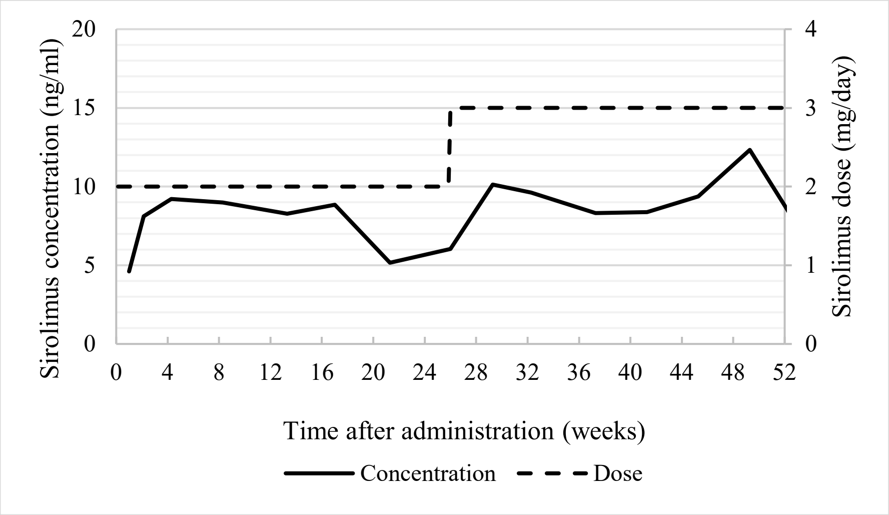

Case 2

a) 22 years old, female, GLA (chest), 150cm, 34.2kg, 1.19m^2

b)

	Pre-treatment	After 12 weeks	After 24 weeks	After 52 weeks
Change of volume of the target lesion		-60.6%	-34%	-30.4%

Evaluation of lesions		PR	PR	PR
MRI	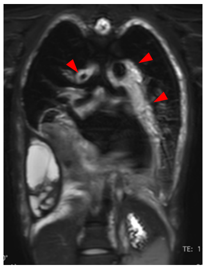	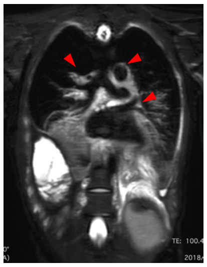	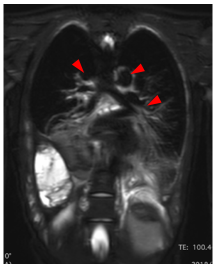	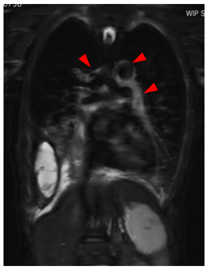
	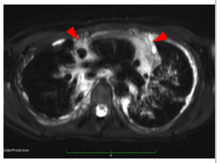	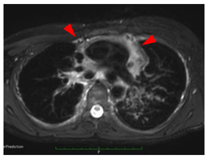	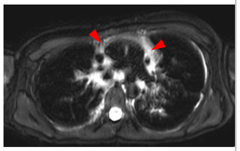	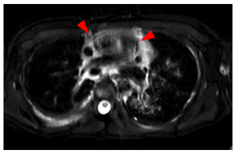

c)

	Pre-treatment	After 12 weeks	After 24 weeks	After 52 weeks
Pleural effusion	+	+ (decrease)	+ (decrease)	+ (decrease)
Ascites	-	-	-	-
Change of other clinical symptoms		Improvement of respiratory disorder		
Platelet counts (/ul)	31.5	27.8	29.3	27.7
Fibrinogen (mg/dl)	416	465	419	350
D-dimer (ug/ml)	31.5	4	1.6	0.9
Bleeding scale	None	None	None	None
Pain scale	4	1	1	0
PS (Karnofsky)	90	90	90	90
QOL (FACT-G)	76.1	77.2	87.0	82.6

d)

	0-12 weeks	13-24 weeks	25-52 weeks
Events (CTCAE grade)	back pain (1), fever (1), musculoskeletal chest pain (1), itching (2), rhinitis (2), diarrhea (2)	diarrhea (1), oral pain (1), diarrhea (1)	burn injury (1), hypertension (2), dizziness (1), acne (1), allergic rhinitis (2)

e)

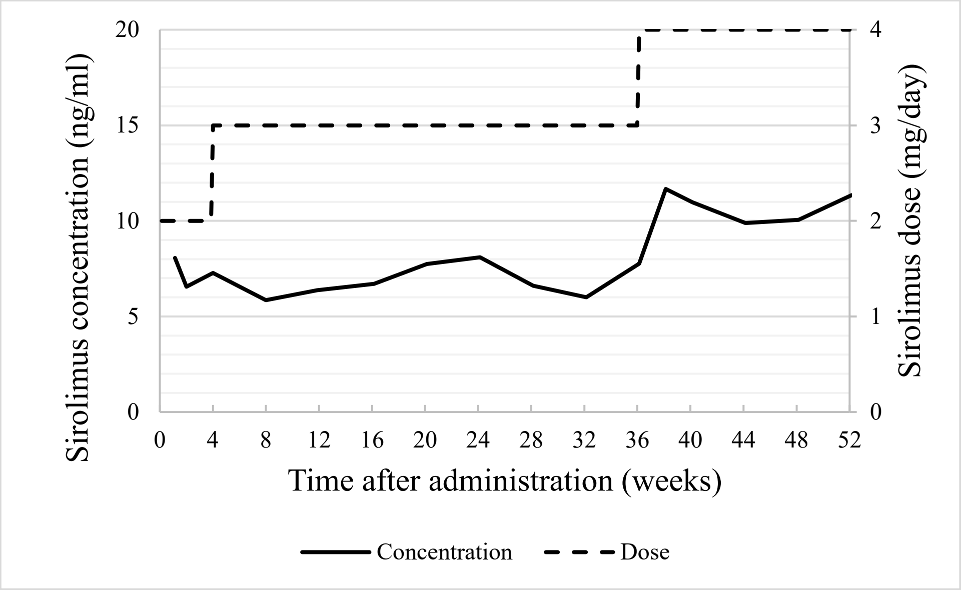

Case 3

a) 5 years old, female, GSD (left chest wall and upper arm), 98cm, 14.6kg, 0.63m^2

b)

	Pre-treatment	After 12 weeks	After 24 weeks	At discontinuation
Change of volume of the target lesion		-62.7%	-60.3%	-59.5%
Evaluation of lesions		PR	PR	PR

MRI	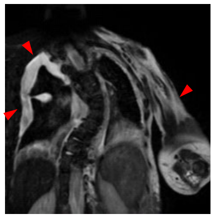	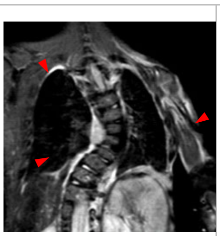	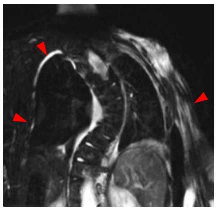	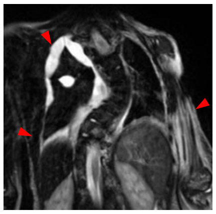
	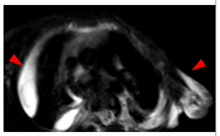	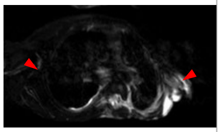	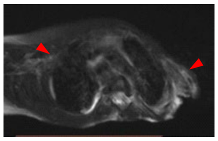	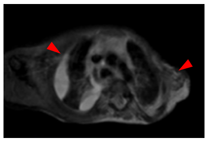

c)

	Pre-treatment	After 12 weeks	After 24 weeks	At discontinuation
Pleural effusion	+	+ (decrease)	-	+
Ascites	N.A.	N.A.	-	N.A.
Change of other clinical symptoms		Improvement of dermatitis		
Platelet counts (/ul)	40.7	36.7	37.9	35.8
Fibrinogen (mg/dl)	169	250	258	190
D-dimer (ug/ml)	40.7	1.7	0.8	24.6
Bleeding scale	None	None	None	None
Pain scale	0	0	0	37
PS (Lansky)	90	90	90	90
QOL (Peds-QL)	87.0	72.3	72.8	50.0

d)

	0-12 weeks	13-24 weeks	25-52 weeks

Events (CTCAE grade)	Bone fracture (2)	Stomatitis (1), gastroesophageal reflux (1), hypertriglyceridemia (3)	Stomatitis (1), contused wound (1), acute hepatitis (3)

e)

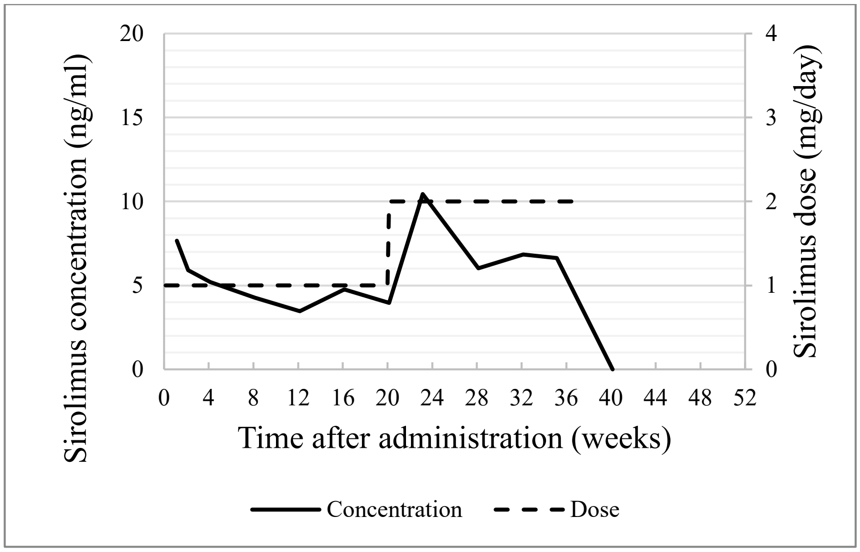

Case 4

a) 24 years old, female, GLA (retroperitoneum), 167cm, 53.3kg, 1.57m^2

b)

	Pre-treatment	After 12 weeks	After 24 weeks	After 52 weeks
Change of volume of the target lesion		-65%	-73.6%	-75.7%
Evaluation of lesions		PR	PR	PR
MRI	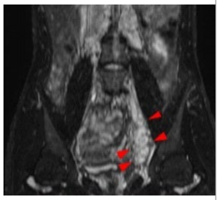	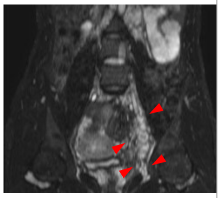	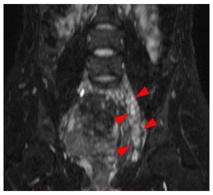	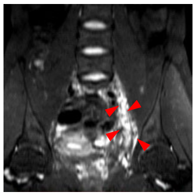

	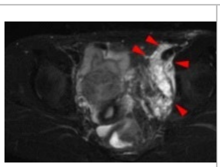	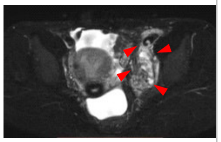	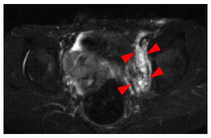	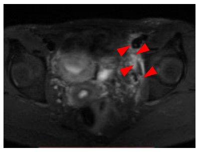

c)

	Pre-treatment	After 12 weeks	After 24 weeks	After 52 weeks
Pleural effusion	-	-	-	-
Ascites	-	-	-	-
Change of other clinical symptoms		Improvement of lymphorrhea and swelling		Improvement of cutaneous lesion
Platelet counts (/ul)	20.3	21.2	21.5	18.5
Fibrinogen (mg/dl)	176	234	201	212
D-dimer (ug/ml)	1.7	7.9	3.6	2
Bleeding scale	Grade1	None	None	None
Pain scale	73	10	60	25
PS (Karnofsky)	80	90	90	90
QOL (Peds-QL)	90.2	80.4	75.0	-

d)

	0-12 weeks	13-24 weeks	25-52 weeks
Events (CTCAE grade)	Diarrhea (1), acne (2), stomatitis (1), fatigue (1), and stomatitis (2)	Upper respiratory inflammation (2) and acne (2)	Stomatitis (1), stomatitis (2), stomatitis (2), stomatitis (2), stomatitis (2), disease resembling influenza (2), edema (1)

e)

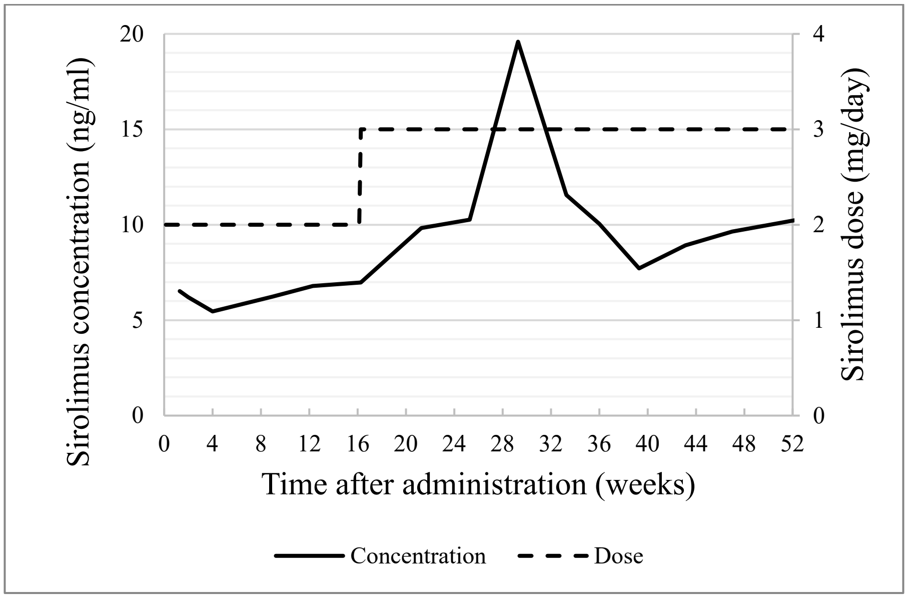

(Visual analog scale of case 4)

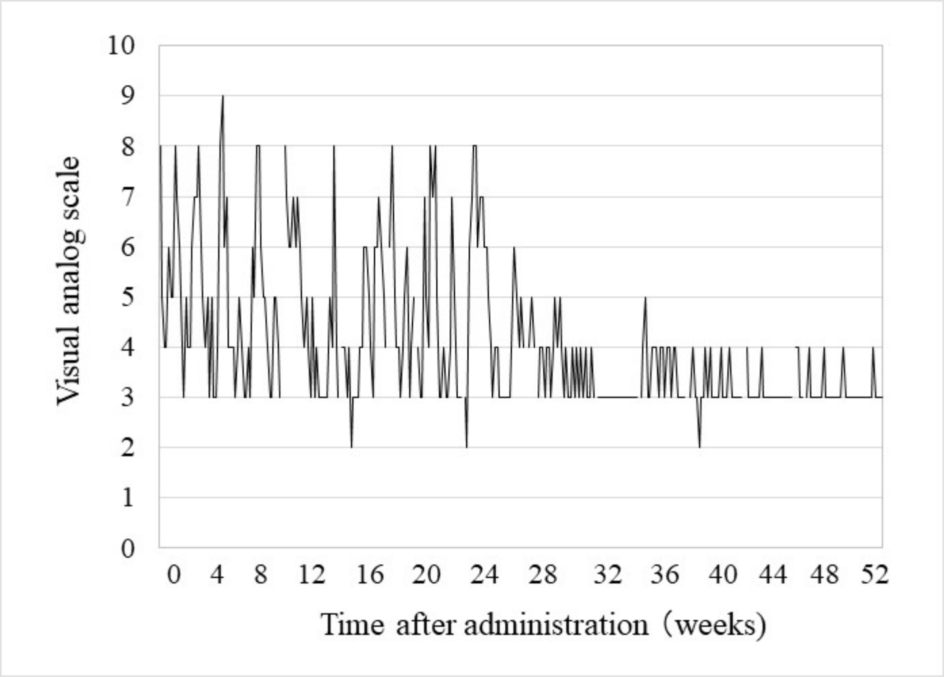

Case 5

a) 26 years old, female, GLA (thoracic), 159cm, 60.1kg, 1.63m^2

b)

	Pre-treatment	After 12 weeks	After 24 weeks	After 52 weeks
Change of volume of the target lesion		12.5%	8.8%	-10%
Evaluation of lesions		SD	SD	SD
MRI	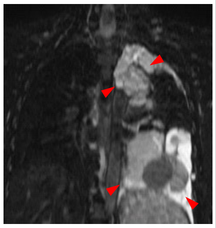	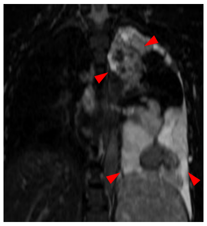	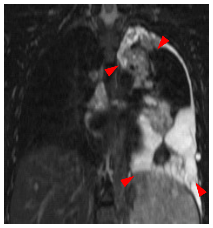	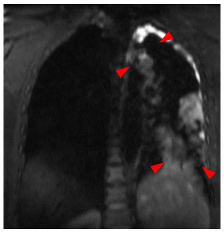
	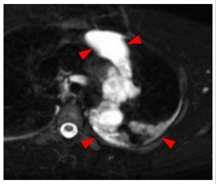	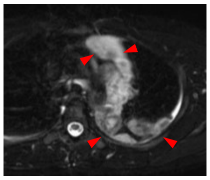	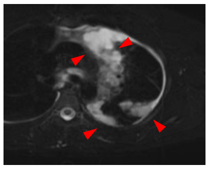	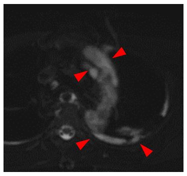

c)

	Pre-treatment	After 12 weeks	After 24 weeks	After 52 weeks
Pleural effusion	+	+	+	+
Ascites	-	-	-	-
Change of other clinical symptoms				
Platelet counts (/ul)	25.4	24.6	18.1	21.7
Fibrinogen (mg/dl)	234	336	362	304
D-dimer (ug/ml)	5.7	9.8	5.4	6.2
Bleeding scale	Grade2	None	Grade2	Grade2
Pain scale	64	69	64	23

PS (Karnofsky)	90	90	90	90
QOL (Peds-QL)	75.0	68.0	65.0	74.0

d)

	0-12 weeks	13-24 weeks	25-52 weeks
Events (CTCAE grade)	Acne (1), stomatitis (2), adenoiditis (2), hypermenorrhea (1), raising of gamma-glutamyl transpeptidase (3), hypermenorrhea (1), nasal hemorrhage (1), and stomatitis (2)	Pharyngitis (2), hypermenorrhea (1), acne (1), raising of alanine aminotransferase (2), hyperlipidemia (2) and hypermenorrhea (1)	Diarrhea (1), nasal hemorrhage (1), sialoadenitis (2), nasal hemorrhage (1), nasal hemorrhage (1), fever (2), diarrhea (2), cough (2), pneumonia (3), hypermenorrhea (1), muscular ache (1), abdominal pain (1), nasal hemorrhage (1), diarrhea (2), dyspepsia (2) and non-cardiac chest pain (1)

e)

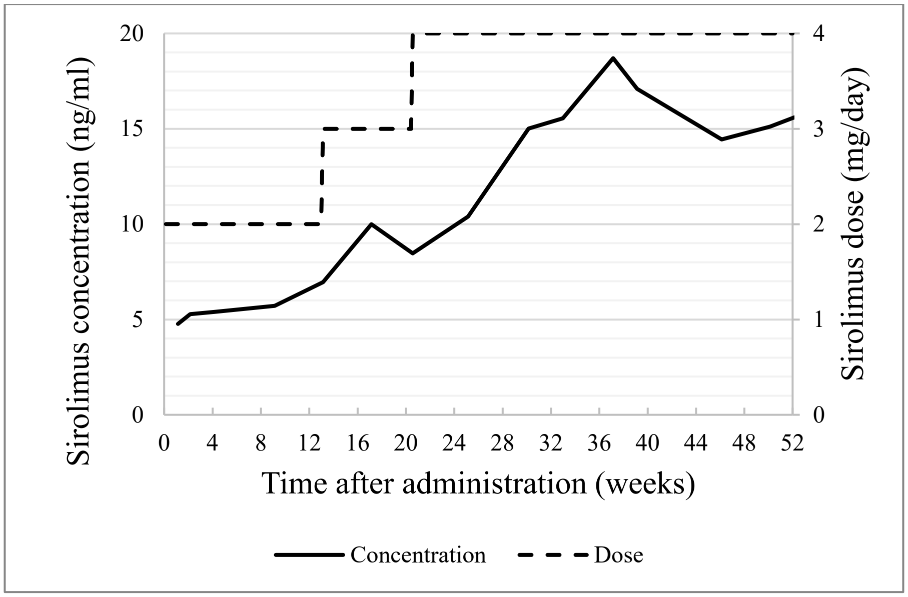

Case 6

a) 14 years old, female, GLA (from lower back to gluteal region), 147cm, 34.3kg, 1.18m^2

b)

	Pre-treatment	After 12 weeks	After 24 weeks	After 52 weeks
Change of volume of the target lesion		-12.2%	-17.1%	-14.4%
Evaluation of lesions		SD	SD	SD
MRI	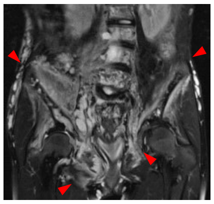	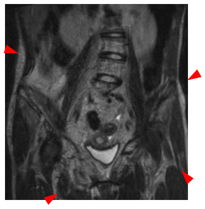	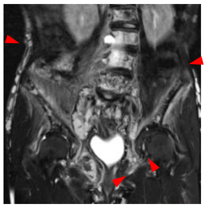	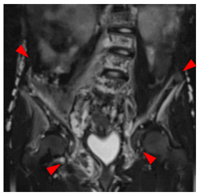
	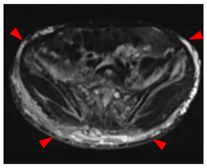	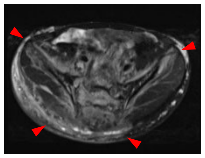	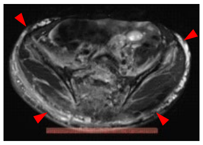	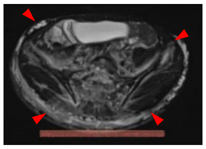

c)

	Pre-treatment	After 12 weeks	After 24 weeks	After 52 weeks
Pleural effusion	-	-	-	-
Ascites	-	-	-	-
Change of other clinical symptoms				
Platelet counts (/ul)	6.5	5.5	6.3	6.5
Fibrinogen (mg/dl)	74	111	-	137
D-dimer (ug/ml)	101.1	63.6	-	53.4
Bleeding scale	None	None	None	None
Pain scale	41	34	53	55

PS (Karnofsky)	90	90	80	90
QOL (Peds-QL)	95.7	-	97.8	97.8

d)

	0-12 weeks	13-24 weeks	25-52 weeks
Events (CTCAE grade)	Muscular ache (1) and eczema (1)	Dehydration (1), pain of skin (1), fever (1), diarrhea (1), abdominal pain (1), and abrasion of skin (1)	Abdominal bloating (3), upper respiratory infection (2), pharyngitis (2), upper respiratory infection (2), pain (3)

e)

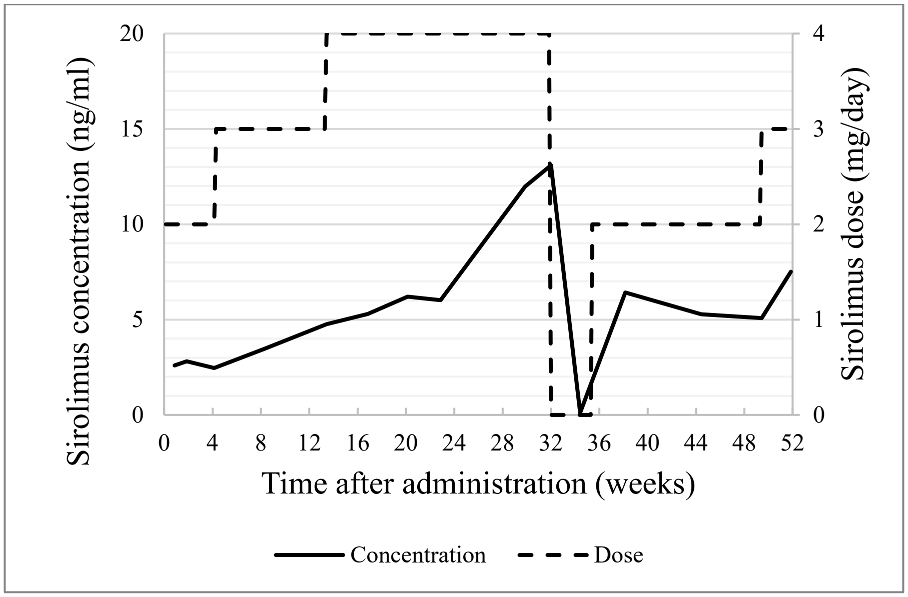

Case 7

a) 10 years old, female, cystic LM (cervical and facial), 127cm, 24.0kg, 0.92m^2

b)

	Pre-treatment	After 12 weeks	After 24 weeks	After 52 weeks
Change of volume of the target lesion		-27%	-46.8%	-40.9%

Evaluation of lesions		PR	PR	PR
MRI	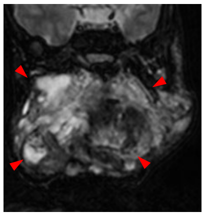	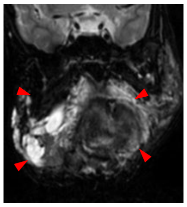	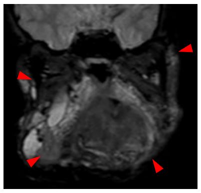	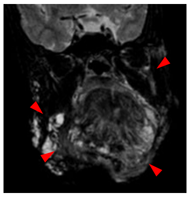
	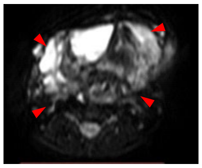	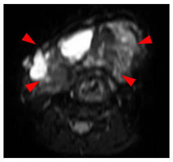	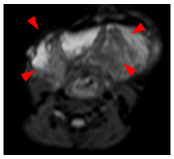	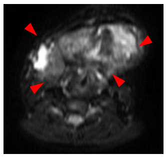

c)

	Pre-treatment	After 12 weeks	After 24 weeks	After 52 weeks
Pleural effusion	-	-	-	-
Ascites	-	-	-	-
Change of other clinical symptoms				
Platelet counts (/ul)	28.4	26.8	36.1	41.4
Fibrinogen (mg/dl)	287	278	421	377
D-dimer (ug/ml)	<0.5	<0.5	<0.5	0.5
Bleeding scale	None	None	None	None
Pain scale	1	0	0	21
PS (Lansky)	90	90	90	90
QOL (Peds-QL)	94.6	95.7	82.6	89.1

d)

	0-12 weeks	13-24 weeks	25-52 weeks
Events (CTCAE grade)	Subcutaneous bleeding (1), erosion (1), carious tooth (2), and stomatitis (1)	Acne (2), skin infection (3), bronchitis (2), and dehydration (2)	Diarrhea (1), skin infection (3), erethism (1) and pneumonia (2)

e)

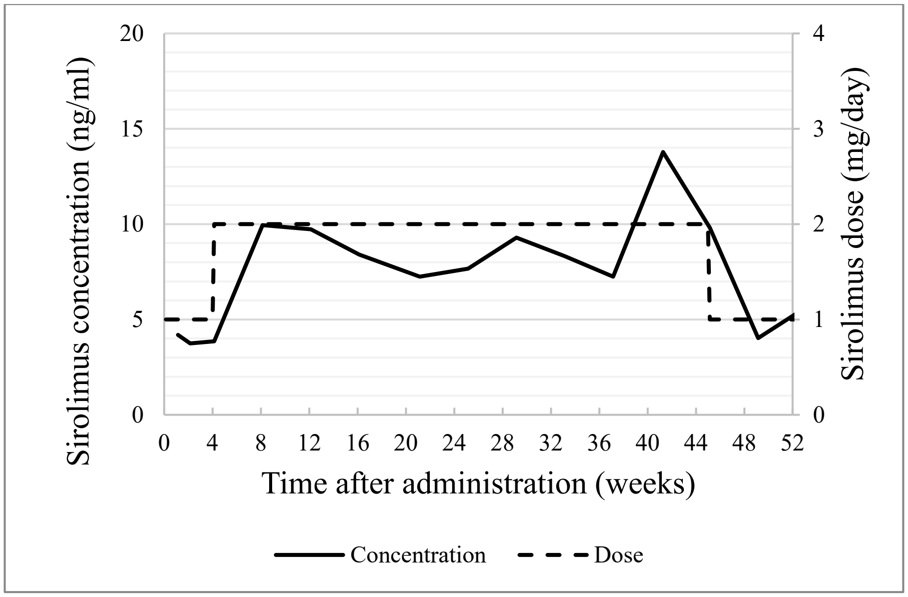

Case 8

a) 9 years old, male, GLA (from left chest to lower back), 136cm, 35.3kg, 1.15m^2

b)

	Pre-treatment	After 12 weeks	After 24 weeks	After 52 weeks
Change of volume of the target lesion		-40.2%	-31.9%	-46.9%
Evaluation of lesions		PR	PR	PR

MRI	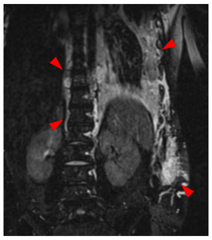	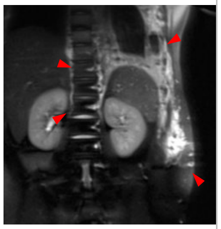	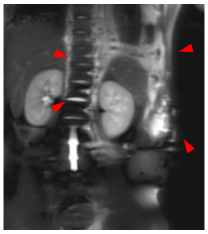	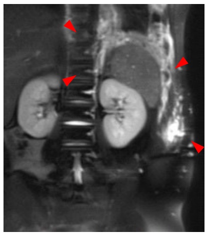
	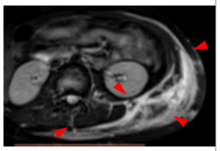	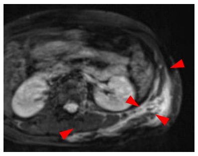	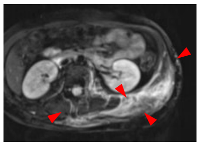	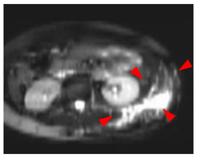

c)

	Pre-treatment	After 12 weeks	After 24 weeks	After 52 weeks
Pleural effusion	+	+	-	-
Ascites	-	-	-	-
Change of other clinical symptoms			Disappearance of pleural effusion	
Platelet counts (/ul)	49.4	32.3	10.6	15.6
Fibrinogen (mg/dl)	132	392	223	343
D-dimer (ug/ml)	19.5	16.1	48.4	22
Bleeding scale	None	Grade1*	None	Grade1*
Pain scale	0	0	60	0
PS (Lansky)	100	90	90	100
QOL (Peds-QL)	95.7	97.8	71.7	97.8

*urinary system

d)

	0-12 weeks	13-24 weeks	25-52 weeks
Events (CTCAE grade)	Stomatitis （1）, abrasion of skin (1), abrasion of skin (1), insect bite (1), fever (2), stomatitis (1), leukocytopenia (2), fever (2), cough (2), hematuria (1), raising of C-related protein (2)	Headache (2), loss of appetite (2), anemia (3), proteinuria (2), pain (2), and low zinc status (2)	Headache (2), stomatitis (1), fever (1), acne (1), and insect bite (1)

e)

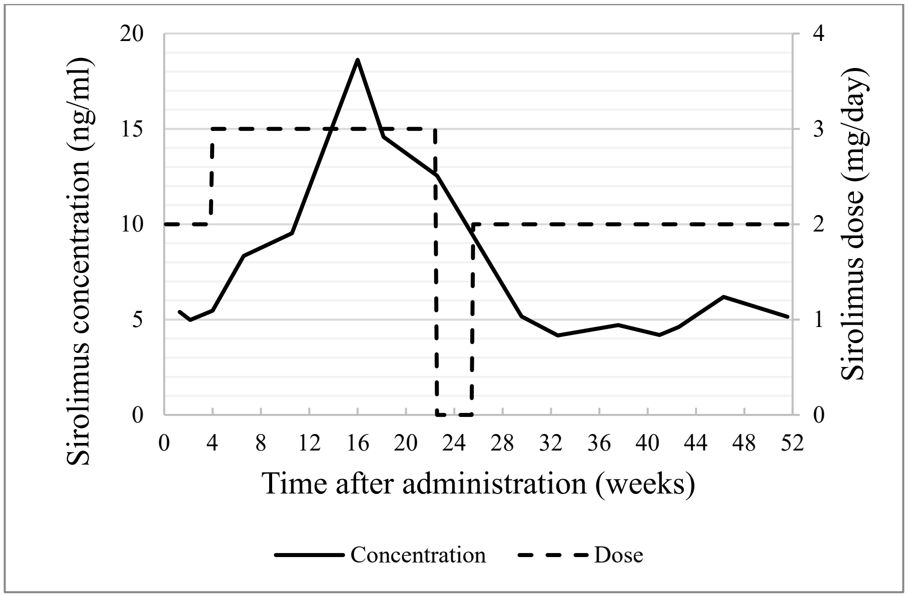

Case 10

a) 23 years old, male, GLA (thoracic), 176cm, 68.6kg, 1.83m^2

b)

	Pre-treatment	After 12 weeks	After 24 weeks	At discontinuation
Change of volume of the target lesion		-24.4%	-24%	1%

Evaluation of lesions		PR	PR	SD
MRI	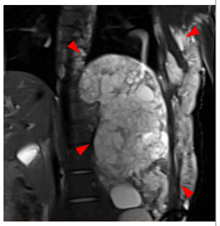	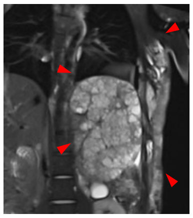	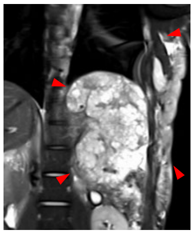	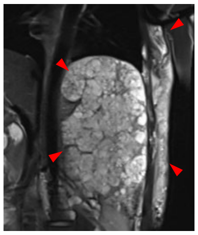
	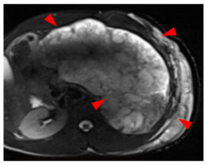	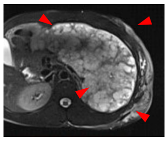	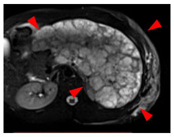	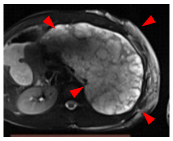

c)

	Pre-treatment	After 12 weeks	After 24 weeks	At discontinuation
Pleural effusion	+	+	-	-
Ascites	-	-	-	-
Change of other clinical symptoms				Worsening of acne
Platelet counts (/ul)	9.1	8.7	10.3	8.2
Fibrinogen (mg/dl)	166	244	214	156
D-dimer (ug/ml)	8.6	13.3	11.4	7.7
Bleeding scale[+]	Grade2	Grade2	Grade2	Grade2
Bleeding scale[$]	Grade1	Grade1	Grade1	Grade1
Pain scale	7	13	22	32
PS (Karnofsky)	90	90	90	90
QOL (Peds-QL)	93.5	-	-	95.7

+Skin

$ Urinary system

d)

	0-12 weeks	13-24 weeks	25-52 weeks
Events (CTCAE grade)	Acne (3), stomatitis (3), and disease of the gums (2)	-	-

e)

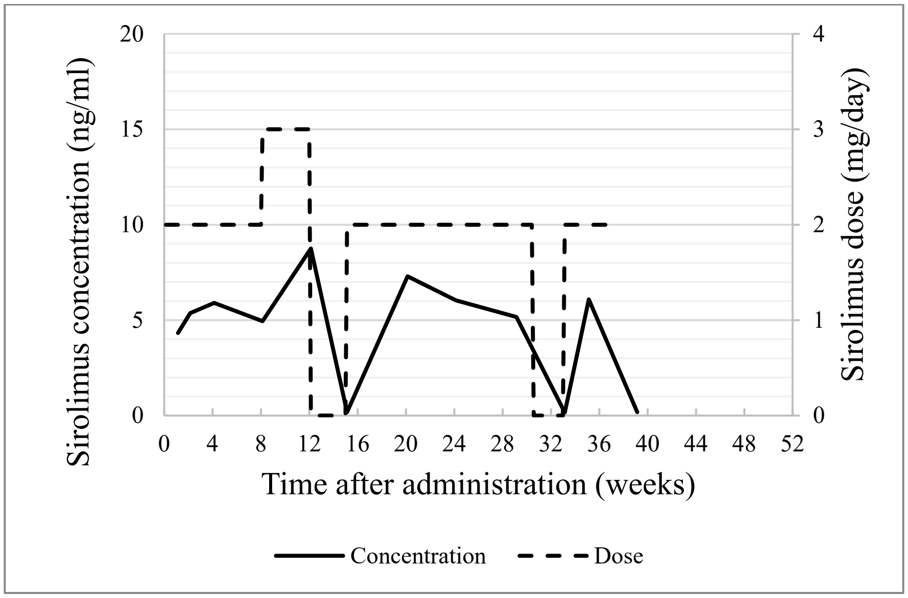

Case 11

a) 24 years old, female, GLA (pelvis and lower extremity), 160cm, 85.3kg, 1.95m^2

b)

	Pre-treatment	After 12 weeks	After 24 weeks	After 52 weeks
Change of volume of the target lesion		0.2%	5.2%	3%

Evaluation of lesions		SD	SD	SD
MRI	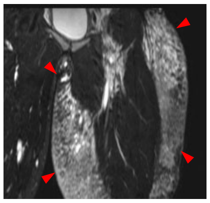	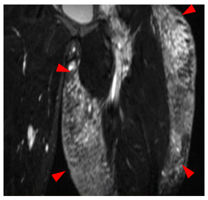	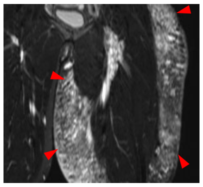	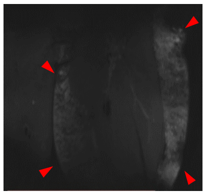
	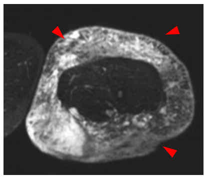	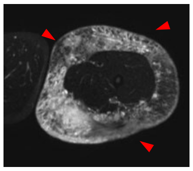	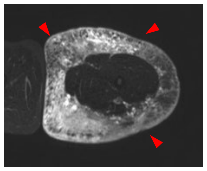	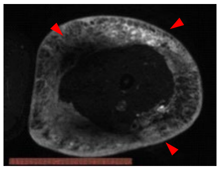

c)

	Pre-treatment	After 12 weeks	After 24 weeks	After 52 weeks
Pleural effusion	+	+	-	-
Ascites	-	-	-	-
Change of other clinical symptoms		Improvement of pain and cutaneous bleeding	Disappearance of lymphorrhea	
Platelet counts (/ul)	17.1	17.4	21.3	21.2
Fibrinogen (mg/dl)	184	300	266	291
D-dimer (ug/ml)	18.8	13.9	29.6	13.9
Bleeding scale[&]	Grade2	None	None	None
Bleeding scale[%]	Grade1	Grade1	Grade1	Grade1
Pain scale	76	43	23	24
PS (Karnofsky)	80	90	90	90

QOL (Peds-QL)	93.5	96.7	89.1	97.8

[&]Skin

[%]urinary system

d)

	0-12 weeks	13-24 weeks	25-52 weeks
Events (CTCAE grade)	Stomatitis （2）, fever (1),<br><br>Eczema (2), pain (1), acne (2), diarrhea (1), stomatitis (2), abdominal pain (1), diarrhea (1), and diarrhea (1)	Hypermenorrhea (1), stomatitis (2), and acne (1)	Nettle rash (2), joint pain (2), stomatitis (1), skin infection (3), oropharyngeal pain (2), stomatitis (1), and stomatitis (1)

e)

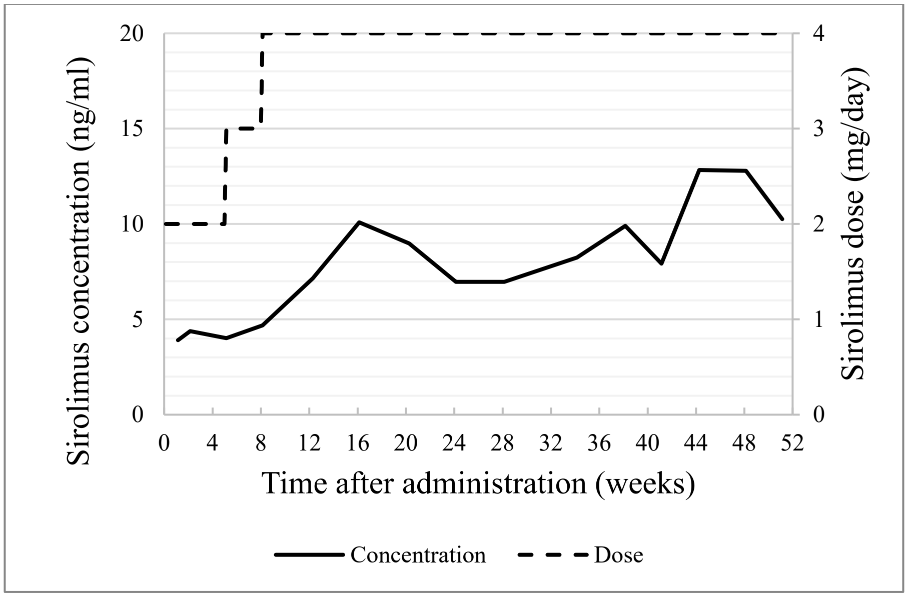

Pre-treatment

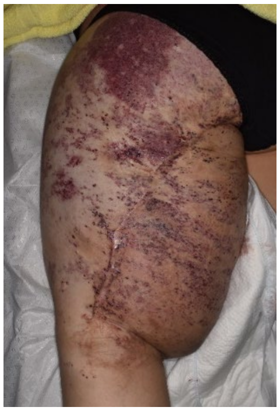

After 24 weeks

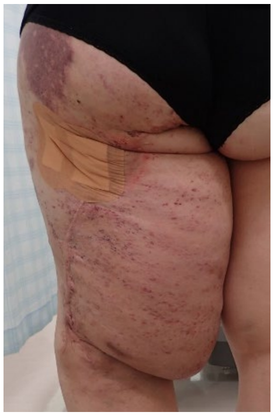

Case 12

a) 3 years old, male, GLA (thoracic and left arm), 98cm, 15.1kg, 0.64m^2

b)

	Pre-treatment	After 12 weeks	After 24 weeks	After 52 weeks
Change of volume of the target lesion		-32.7%	-71.3%	-63.1%
Evaluation of lesions		PR	PR	PR
MRI	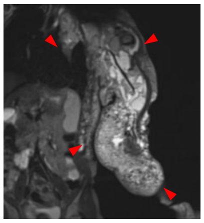	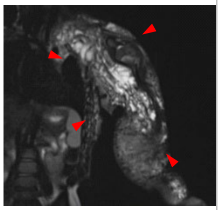	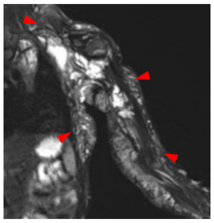	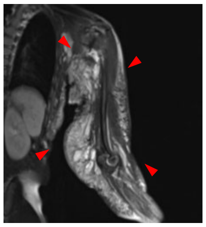
	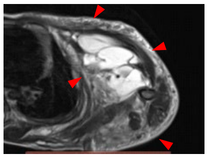	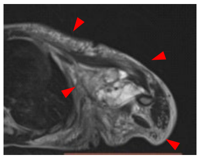	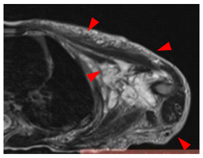	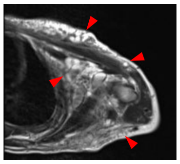

c)

	Pre-treatment	After 12 weeks	After 24 weeks	After 52 weeks

Pleural effusion	-	-	-	-
Ascites	-	-	-	+
Change of other clinical symptoms		Improvement of skin bleeding		
Platelet counts (/ul)	42.4	45.9	47.2	45.7
Fibrinogen (mg/dl)	205	234	246	277
D-dimer (ug/ml)	9.6	5.8	0.8	4.6
Bleeding scale[!]	Grade1	None	None	None
Pain scale	-	-	-	-
PS (Lansky)	100	100	100	100
QOL (Peds-QL)	85.7	86.9	89.3	82.1

!Skin

d)

	0-12 weeks	13-24 weeks	25-52 weeks
Events (CTCAE grade)	Fever (1), upper respiratory infection (2), elevation of cholesterol and low density lipoprotein level (1), skin infection (1), insect bite (2), stomatitis (1), rhinorrhea (2), and insect bite (2)	Infectious enteritis (2), Infectious enteritis (2), dermatitis (2), cutaneous ulceration (2), and bronchitis (1)	Papule (1), conjunctivitis (2), upper respiratory infection (2), conjunctivitis (2), and upper respiratory infection (2)

e)

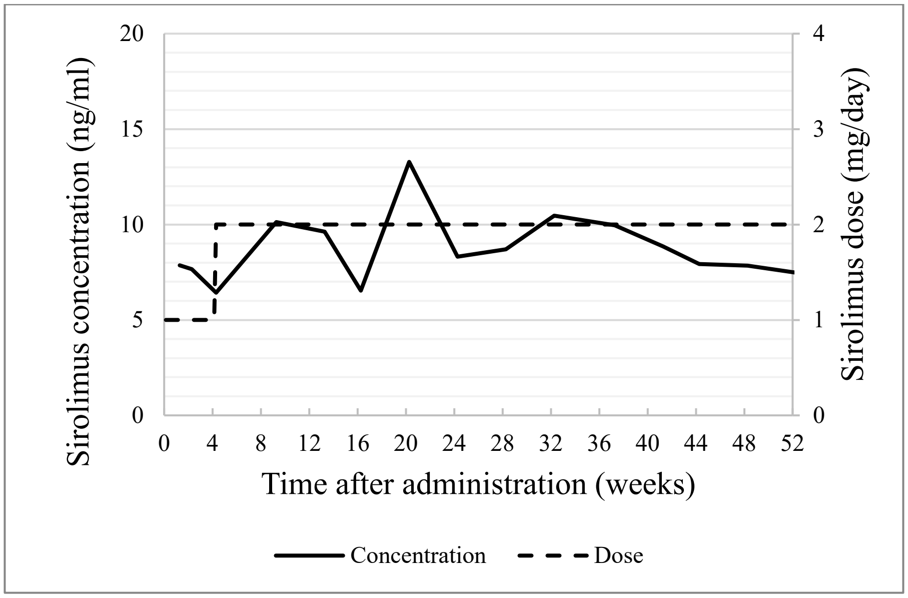
Sirolimus concentration (ng/ml)
Sirolimus dose (mg/day)
Time after administration (weeks)
0 4 8 12 16 20 24 28 32 36 40 44 48 52
0 5 10 15 20
0 1 2 3 4
Concentration
Dose
